# The four Rs and crystal structure analysis: reliability, reproducibility, replicability and reusability

**DOI:** 10.1107/S1600576722007208

**Published:** 2022-08-24

**Authors:** John R. Helliwell, Chiara Massera

**Affiliations:** a Department of Chemistry, University of Manchester, Manchester M13 9PL, United Kingdom; b Dipartimento di Scienze Chimiche, della Vita e della Sostenibilità Ambientale, Università di Parma, Viale delle Scienze 17/A, Parma 43124, Italy; Wilfrid Laurier University, Waterloo, Ontario, Canada

**Keywords:** trust, reliability, reproducibility, replicability, reuse

## Abstract

Within science, of which crystallography is a key part, there are questions posed to all fields that challenge the trust in results. The US National Academies of Sciences, Engineering and Medicine published a thorough report in 2019 on the *Reproducibility and Replicability of Science*: replicability being where a totally new study attempts to confirm if a phenomenon can be seen independently of another study. Data reuse is a key term in the FAIR data accord [Wilkinson *et al.* (2016). *Sci. Data*, **3**, 160018], where the acronym FAIR means findable, accessible, interoperable and reusable. In the social sciences, the acronym FACT (namely fairness, accuracy, confidentiality and transparency) has emerged, the idea being that data should be FACTual to ensure trust [van der Aalst *et al.* (2017). *Bus. Inf. Syst. Eng.* **59**, 311–313]. A distinction also must be made between accuracy and precision; indeed, the authors’ lectures at the European Crystallography School ECS6 independently emphasized the need for use of other methods as well as crystal structure analysis to establish accuracy in biological and chemical/material functional contexts. The efforts by disparate science communities to introduce new terms to ensure trust have merit for discussion in crystallographic teaching commissions and possible adoption by crystallographers too.

## Abstract 2

Across the sciences, generic monitors of trust in results from science are being introduced. As educators and researchers, the authors consider these terms within the context of biological and chemical crystal structure analyses.

## 1. Introduction

Trust in science is generally assumed by scientists and is yet ever more under scrutiny if there are failures. From the article *Trust in Science* (Barber, 1987 ▸):

‘TRUST is an essential constituent of all social relationships and all societies.

One sense of trust refers to an expectation or prediction that an assigned or accepted task will be competently performed. We trust, in this sense, that a person who is acting in a particular role or a particular capacity will do so at a reasonably expected level of proficiency. This meaning of trust is important…in contemporary societies where there is such a vast accumulation of knowledge and technical expertise based on that knowledge. Scientists very much expect that a scientist who has the qualifications adjudged necessary to be a scientist can be trusted in this sense.

A second meaning of trust is the reposing of fiduciary obligations and responsibilities in an individual or on an individual. We trust that the person will fulfil his duty…and that they will place the obligations which are by tradition inherent in their role…above their own immediate interest or anticipated advantage… Scientists very much expect that a qualified scientist can be trusted in this sense too… Trustfulness, trustworthiness, trust in both senses are indispensable to the growth of scientific knowledge.

These two forms of trust are quite distinct from each other. This is certainly the case in science.’

So, the apprentice scientist must learn to be trustworthy in both senses. Our article, we hope, can be regarded as a guide to such an apprentice. We suggest that training courses for young crystallographers should develop many of the things we identify in our article (*i.e.* fostering a deeper understanding of the limitations as well as the potential of their data). We consider the Naples Crystallographic Information Framework Fiesta (https://www.iucr.org/resources/cif/comcifs/cifiesta-2019), organized by the Italian Crystallographic Association and the International Union of Crystallography, an exemplar of such a course.

**Figure 1:**
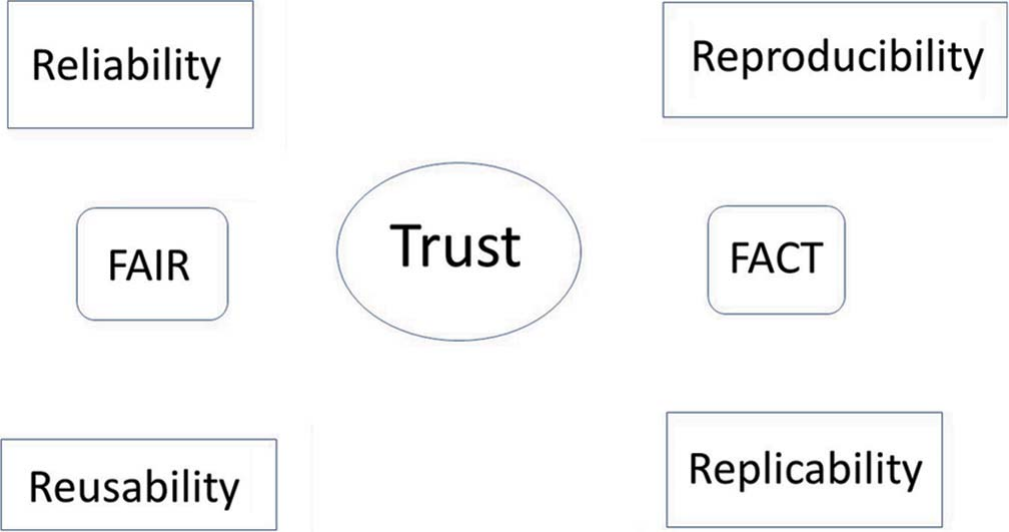
Trust in science is built up from different facets, which we detail in this article. These keywords that surround the central word ‘trust’ include our usual crystallographic community word ‘reliability’. Other science communities and policy bodies have emphasized other keywords to ensure ‘trust’ in science. We think each has merit and could usefully assist the crystallographic community policies such as in journal notes for authors, as well as how we engage with the public and students.

More and more, in all the sciences, the aim is ease of reuse of data to assess the reproducibility of a study by providing access to the data underpinning a publication. This allows the reader of a study to understand published research results through their own eyes. From the first crystal structure analysis, our tradition in crystallography has been to include or attach our data (Bragg, 1913 ▸). Indeed, the field of crystallography is widely regarded as a leader in attaching the article narrative to the underpinning data. For this we are widely admired, as demonstrated by the awarding of the International Science Council CODATA Prize in 2014 to Professor Sydney Hall (see https://codata.org/codata-prize-2014-awarded-to-professor-sydney-hall/). The aim of the present educational article is to set crystallographers’ monitors of correctness into the more general scientific context. The US National Academies of Sciences, Engineering and Medicine published a thorough report in 2019 ▸ on the *Reproducibility and Replicability of Science*: replicability being where a totally new study attempts to substantiate if a phenomenon can be seen independently of another study. We hope our article could assist with a taught course on ‘trust in science and the role of crystallography’, whose learning outcomes would include an informed understanding of what we term the four Rs of crystal structure analysis: reliability, reproducibility, replicability and reusability. A students’ discussion seminar on ‘trust in science and the role of crystallography’ could explore firstly the domain of crystallography and then the history of science examples presented in the book by Oreskes (2019 ▸), which one of us has reviewed from the point of view of a crystallographer (Helliwell, 2019 ▸). The assessment of participants who had attended a taught course or seminar would most likely involve an essay-type question such as ‘critically assess the role of crystallography in effecting trust in science as a whole’. In the subsequent sections we describe from a crystallographer’s point of view how we can define trust in what we do, which we illustrate with a simple infographic (Fig. 1 ▸).

The fairly new acronyms FAIR and FACT have the following meanings: FAIR means findable, accessible, interoperable and reusable and is a general term in data science. FACT means fairness, accuracy, confidentiality and transparency and has emerged from the social sciences for data. Whereas FAIR looks at practical issues related to the sharing and distribution of data (Wilkinson, 2016 ▸), FACT focuses more on the foundational scientific challenges (van der Aalst *et al.*, 2017 ▸). In crystallography, the requirement for FAIR data is satisfied by our databases for processed diffraction data and their derived molecular models. van der Aalst *et al.* (2017 ▸) neatly explained their concepts as follows:

‘Q1 fairness: data science without prejudice – how to avoid unfair conclusions even if they are true?

Q2 accuracy: data science without guesswork – how to answer questions with a guaranteed level of accuracy?

Q3 confidentiality: data science that ensures confidentiality – how to answer questions without revealing secrets?

Q4 transparency: data science that provides transparency – how to clarify answers so that they become indisputable?’

These questions stimulate new thinking in our minds as crystallographers. Confidentiality is the one concept that is truly the domain of social sciences involving personal or medical data. That said, pre-publication peer review must involve confidential scrutiny of an article with underpinning data involving only an editor and their chosen referees.

Coming back to our own specific domain, the procedure for a crystal structure analysis that is generally used today involves the following steps. The first is crystallization, followed by diffraction data collection, and then a solution to the phase problem is sought. Next, a difference Fourier electron density map is calculated to locate any missing atoms or indicate disordered moieties. Finally, a molecular model refinement is undertaken, with *checkCIF* or PDB report alerts for use by the crystallographer as core guidance (see *e.g.* Giacovazzo *et al.*, 2011 ▸). The crystallography community has developed a distinct crystallographic information framework (CIF) of clear ontologies within a CIF file (see *e.g.* Hall & McMahon, 2016 ▸). The International Union of Crystallography has a Committee for the Maintenance of the CIF Standard (https://www.iucr.org/resources/cif/comcifs), established in 1993. Central to this approach is a check of the CIF file; *checkCIF* reports on the consistency and integrity of crystal structure determinations reported in CIF format. Similarly, any Protein Data Bank deposition involves an extensive advisory PDB validation report (https://www.wwpdb.org/validation/validation-reports) assessing numerous indicators of correctness against the processed diffraction data and expected molecular geometry values.

## 2. Reliability

In the history of crystal structure analysis, a major methodological transition to calculate precision indicators of the atomic positions and their displacement parameters was the introduction by Hughes (1941 ▸) of least-squares model refinement against diffraction data. In terms of technology, Hughes (1941 ▸) described using an ‘International Business Machines Co. Tabulator using the Hollerith punched card system’ instead of the manual Beevers Lipson strips. Hughes’ (1940 ▸) discussion of reliability involved the measured intensities but not the molecular model. A. J. C. Wilson’s (1950 ▸) article focused on the molecular model and opened with ‘The reliability index 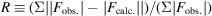 is widely used as a test of the quality of a structure determination.’ The reliability index can be called more simply the least-squares residual, which is then not judgemental. Cruickshank (1960 ▸) discussed (i) the requirements necessary for determining bond lengths crystallographically within an error limit of 0.01 Å and (ii) the required precision for X-ray diffraction intensities and (iii) gave a simple approximate formula relating the residual *R* to the coordinate estimated standard deviation. Although reliability is implicit in the considerations discussed, Cruickshank does not explicitly use the word, in contrast to Wilson (1950 ▸). Also, whilst Hughes (1940 ▸, 1941 ▸) tabulated the measured structure factor amplitudes and the corresponding values calculated from the molecular model, an overall residual was not calculated. Hughes (1941 ▸) emphasized the practical details of the calculation for the melamine crystal structure, which comprised nine non-hydrogen atoms, as follows:

‘The cards were punched, verified, and the normal equations produced in slightly less than two days. The resulting normal equations consisting of eighteen simultaneous equations in the eighteen parameters were solved by an iteration method in about four hours.’

Interest in molecular model refinement was evidently stirred by the Hughes (1941 ▸) paper, which was clearly a breakthrough in spite of the limited calculational technology of the time, and other variants followed. The Fourier method developed by Booth (1945 ▸, 1946 ▸, 1947 ▸) involved corrections to atomic parameters in real space based on the difference Fourier map calculation. The relationship between the Fourier and least-squares methods was discussed by Cruickshank (1952 ▸).

The model refinement of biological macromolecules presented different challenges. A summary article (Murshudov *et al.*, 1997 ▸), which links to those early calculation methods, states

‘It was recognized in the 1960’s that macromolecular refinement posed special problems. There were too few observations to refine the atomic parameters using least-squares minimization alone, and the calculation of the structure factors and derivatives from such a large number of coordinates challenged the computing resources available.’

A key help, to add observations aside from the diffraction data, was the availability of dictionary values of bond distances and angles from chemical crystallography that could act as restraints (Konnert, 1976 ▸; Konnert & Hendrickson, 1980 ▸). Murshudov *et al.* (1997 ▸) considered the reliability of coordinates within a maximum likelihood formalism for the refinement. The assumption that different parts of a structure might have different errors was considered. Cruickshank (1999 ▸) introduced the diffraction precision index to provide a measure of the overall precision of the coordinates of a protein crystal structure based on the processed X-ray diffraction data. This was extended to non-bonded individual atoms by Gurusaran *et al.* (2012 ▸) and Kumar *et al.* (2015 ▸). A new measure of agreement of the molecular model to the protein crystal diffraction data was *R*_free_ (Brünger, 1992 ▸), where a 5–10% subset of reflections are excluded from the refinement to secure an unbiased model. Interestingly, chemical crystallography has not introduced the *R*_free_ statistical indicator. Another measure of reliability is the correlation coefficient. In macromolecular crystallography, the quality of the anomalous differences, for example, can be assessed by splitting a diffraction data set into two halves and calculating the correlation coefficient between the anomalous differences within those two half data sets. The various statistical indicators used by macromolecular crystallographers are described in detail by Einspahr & Weiss (2012 ▸). In chemical crystallography, aside from the *R* factor, various other parameters are checked such as resolution, redundancy, weighting parameters, goodness of fit and wR2; the differences between *F*_obs_ versus *F*_calc_ are also checked as a further validation tool. Note that the presence of systematic errors and concerns about the goodness of fit have been expressed in a data review of a large number of chemical crystal structures (Henn, 2019 ▸).

## 3. Reproducibility

The way that crystallographers have included or linked their article narrative to their derived molecular coordinates, and then also to their diffraction data when the digital storage capacity of the hardware expanded, has allowed a check on the reproducibility. In judging the data underpinning an article, the reader assesses the workflow that the authors have followed. There are numerous steps and various software programs that can be used. A sensible view will need to be taken of the author’s steps, which may not be the preferred steps that the reader would have taken. There may be a variance that can be allowed. Outside that variance, however, errors can be determined. Deciding how much variance is allowable is not always easy. We must address several questions: Can equivalent crystal structure analysis workflows be allowed a variance of results, such as the molecular model coordinates, within this concept of reproducibility? Are macromolecular and chemical crystallography different in this regard?

Helliwell (2022 ▸) addresses this in detail for macromolecular crystallography; one clear example is when a researcher must decide whether to include a given bound water molecule in a molecular model or not. This is an important consideration in macromolecule ligand binding, which is a topic of considerable importance both in structure-based drug design and when considering the thermodynamics of ligand binding across similar types of ligands, such as in calorimetry measurements (see *e.g.* Bradbrook *et al.*, 1998 ▸). There are, in practice, a variety of criteria with no clear standards.

In protein crystallography, the PDB-REDO project based in Utrecht (Joosten *et al.*, 2009 ▸) is a useful initiative because data analysis workflows and software are continually developing. A direct comparison of the original PDB-deposited model and the current PDB-REDO model illustrates the range of variances that are possible. These opportunities to explore variances will expand with the growing trend towards raw diffraction data archiving. This has resulted in the new IUCr Journals policy led by the IUCr Commission on Biological Macromolecules to require that a digital object identifier (DOI) for the underpinning raw diffraction data for a new structure and for raw data processing software papers must now be quoted in the publication as well as having the PDB deposited files.

By contrast the chemical crystallography community has been less interested in archiving raw diffraction data, except in selective cases of challenging diffraction (see *e.g. *the workshop linked with the IUCr’s 2021 Prague Congress; https://www.iucr.org/resources/data/commdat/prague-workshop-cx). There is, though, a greater homogeneity of crystal quality in chemical crystallography, which has guaranteed a consistently good diffraction resolution limit.

## 4. Replicability

Let us first consider the opposite of replicability, namely falsification. Does science advance most by consensus (repeated replicability) or by ‘falsification’? In the philosophy of science these extremes are firmly advocated [respectively, by Oreskes (2019 ▸) and Popper (2002 ▸)]. Falsification as a rationale initially has its attractions for the scientific process, but if a result is wrong because of misinterpretation or because a false protocol or workflow was used then the analysis must simply be redone. Replicability to prove a result seems a more robust test than falsification. We provide two examples, one from each of our respective research fields.

In a collaboration involving several laboratories in London and Manchester, we determined the crystal structure of the lobster carapace component responsible for the blue–black colour, namely β-crustacyanin, using a crystal that itself was blue. The colour of the protein in its solution was the same blue, by eye. In addition, the measured UV–Vis spectrum quantified the solution colour. We also measured the small-angle X-ray scattering (SAXS) of the β-crustacyanin in solution, and the calculated SAXS curve from the cryo-crystal structure model was an excellent fit. These results are described by Chayen *et al.* (2003 ▸). This example also illustrates how accuracy is reached using multiple methods. Each method can be individually precise, but taken together accuracy is realized. The point here is that an individual method has both random errors in its measurements and systematic errors. A least-squares fit to the measured data can minimize the impact of random errors for each method and yield precision but cannot circumvent the systematic errors in the method. The latter can only be avoided by harnessing two or more other methods. In the lobster coloration study, we combined X-ray crystallography, UV–Vis spectroscopy and SAXS. The measurements were performed on different sample states that were each blue: a cryo-frozen crystal at 100 K was used for X-ray crystallography, and the UV–Vis spectroscopy and SAXS measurements of the solutions were made at room temperature. The by-eye observations of the crystal colour, the solution colour and the lobster carapace itself we must call qualitative spectral observations as opposed to the quantitative UV–Vis spectroscopy, but nevertheless they are emphatic evidence that we had taken measurements of the right thing. The combination of methods, and their repeated replicability, confers accuracy in the results.

An analogous conclusion can be drawn from another example taken from the field of supramolecular chemistry. In a collaboration with the University of Eastern Finland, a single-crystal-to-single-crystal transformation was reported, triggered by guest exchange in a tetra­phospho­nate cavitand (Massera *et al.*, 2011 ▸). First, the cavitand was shown to be selective towards methanol when single crystals of the water/acetone solvate exposed to the alcohol could uptake it while releasing water and acetone molecules. Secondly, the inherent selectivity of the cavitand was demonstrated by guest-exchange experiments monitored by ^31^P NMR spectroscopy in solution. Finally, the existence of water and methanol complexes of the cavitand in the gas phase and their relative kinetic stability were monitored by ESI-MS experiments. Hence, this example shows that the replacement of water with methanol is controlled by the molecular-recognition properties of the host component in all three phases. The combined use of different techniques ensures that the phenomenon described is true and has been modelled in an accurate way.

## 5. Reusability

The FAIR principles specifically include reusability, *i.e* the ‘R’ of FAIR. The opportunities for crystallographic data reuse rely on the various crystallographic databases (Hall & McMahon 2016 ▸; Bruno *et al.*, 2017 ▸). They are regarded as an exemplar in science, as measured, for example, by the series of lectures and workshops held in April 2022 organized by the US National Committee on Crystallography (USNCCr), the US National Academies of Science, Engineering and Medicine, and the US National Institute of Standards and Technology (NIST) on the crystallographic and structural databases. Details, including some recordings, are available at https://www.nationalacademies.org/our-work/exploring-structural-database-use-in-crystallography-a-usnccr-workshop-series).

Crystallographic raw data are now also being archived by researchers, which is possible because of the colossal expansion of digital archives. This is an important development for crystallography and crystallographers in satisfying the FAIR principles (Terwilliger, 2014 ▸). The worldwide Protein Data Bank and the Cambridge Structural Database now have places in a deposition that allow citation by the depositor of the DOI to a raw diffraction data set. More explicitly, the Protein Data Bank Japan (PDBj) has launched its own X-ray Diffraction Archive (XRDa) to allow depositors to archive their raw diffraction data sets as well as depositing their processed diffraction data and derived molecular models in the PDBj itself.

A wide variety of crystallography case studies documenting the importance of data reusability, now including the archived raw diffraction data, are described in the article by Helliwell *et al.* (2017 ▸). During the recent Covid-19 pandemic, crystallographers have been able to undertake data reuse to effect improvements of molecular models of individual Covid-19 protein crystal structures [Aragao *et al.*, 2020 ▸ (this is just one example of around 50 such raw data set depositions from this research team); Fraser Lab & Collaborators, 2020 ▸; Jaskolski *et al.*, 2021 ▸; https://github.com/thorn-lab/coronavirus_structural_task_force).

In Section 7 we describe cases where fabricated crystal structures have been reported. Their discovery, of course, was due to data reuse being possible, because the articles concerned had to be accompanied by their underpinning (albeit fabricated) data.

## 6. Accuracy (combining individual precise methods to realize accuracy)

As exemplified in Section 4, combining crystal structure analysis with other complementary techniques which can corroborate one another can ensure accuracy. It not only validates the trustworthiness of the structural model but helps to shed light on the correlation between structure and function, on the dynamic behaviour of materials, and on their possible practical applications. Helliwell (2021 ▸) provides a wide variety of further examples.

Though NMR, mass spectrometry and computational modelling are complementary techniques for both chemical and biological crystallography, we have chosen to discuss these methods separately because they are applied in different ways.

### 6.1. Chemical crystallography

The complementary methods available to the chemical crystallographer are many, and it would be beyond the scope of this paper to list them all. It is, however, useful to acknowledge their importance in structural chemistry by providing some selected examples taken from the literature. In particular, they involve the use of (i) thermal analysis, (ii) spectroscopy, (iii) gas chromatography–mass spectrometry (GC-MS) and (iv) computational methods.

(i) Thermal analysis involves measuring the changes of various physical properties of a sample against variations of the temperature. Specifically, differential scanning calorimetry measures the heat flow into or out of the sample against that of a reference during a thermal cycle. It allows one to study thermodynamic processes, phase changes and transitions. A good example of its use can be found in a paper by Nikolayenko *et al.* (2018 ▸), which describes the behaviour of a porous halogen-bonded framework that can adapt dynamically upon uptake of different gases. In this work, pressure-gradient differential scanning calorimetry was used to determine the gas-specific onset pressures of the structural transformations to obtain a mechanistic insight into the breathing behaviour of the framework.

(ii) Spectroscopy studies the interaction of matter with different types of radiation. Fourier transform (FT)–IR, Raman, UV–Vis and NMR spectroscopy are some of the most routinely used techniques in chemistry laboratories. Their role in providing complementary information in solution has already been exemplified in Section 4 for UV–Vis and NMR. Solid-state NMR is the principal technique employed in the field of NMR crystallography and can provide structural and dynamic information on various types of solid materials (Ripmeester & Wasylishen, 2013 ▸; Bryce, 2017 ▸). FT–IR and Raman both involve the study of the interactions of radiation with the molecular vibrations of a sample and are generally associated with the bond strength between atoms in molecules. Moreover, they can help clarify problems that cannot be solved solely through X-ray diffraction: see for instance the work of Brudler *et al.* (2001 ▸), Baumgartner *et al.* (2021 ▸) and Cappuccino *et al.* (2018 ▸). This last paper, for instance, is an example in which Raman spectroscopy was used to identify the conformational polymorphs of a series of quaterthio­phene derivatives. By detecting the spectroscopic differences between *syn*–*anti*–*syn* and *anti*–*anti*–*anti* conformers, the authors used these as a means of validation for structures obtained through X-ray powder diffraction.

(iii) GC-MS is a powerful tool for the identification of different species in a mixture and, incidentally, the combination of these two complementary techniques enhances the accuracy of the final result. The contribution of GC-MS in crystallography is particularly evident when dealing with porous materials (such as metal, covalent and supramolecular organic frameworks) that are filled with guests that are not always clearly identifiable (for instance because of disorder) through diffraction analysis. On a subtler level, this technique can be potentially used to assess the binding strength of the guests inside the pores, if a correlation can be established to their preferential release in response to an external stimulus. This is what has been described in a paper by Balestri *et al.* (2021 ▸), in which the authors have analysed the host–guest interactions of eugenol and thymol inside a zinc-based metal organic framework (MOF). After investigating the supramol­ecular interactions responsible for the uptake of the guests inside the pores by means of single-crystal X-ray diffraction, the authors performed controlled guest-release studies at different temperatures using static headspace GC-MS analyses, which revealed the stronger interaction of eugenol with the pores of the MOF.

(iv) One of the benefits of computational chemistry is the ability to generate data that can be used to rationalize and possibly predict the behaviour of a system. It is regularly used in solid-state analyses for modelling, for crystal structure prediction and to assess the energy of crystal structures. Moreover, it is an essential tool in quantum and NMR crystallography. Its use to ensure accuracy of a crystallographic model is exemplified in a paper by McConville *et al.* (2020 ▸), reporting the phase diagram of a cocrystal of benzene and aceto­nitrile. While investigating the solid-state structure of a specific region of the diagram with variable-temperature X-ray powder diffraction, the authors obtained an aceto­nitrile:benzene cocrystal in a 1:3 ratio, which was solved in the trigonal space group *R*3. An alternative possible solution of the structure was in space group *R* 3 but with disordered aceto­nitrile molecules in the crystal packing. The correctness of the refinement was proved by performing an energy optimization of the two possible arrangements (centrosymmetric and noncentrosymmetric) of a selected cluster of molecules. Only the noncentrosymmetric cluster reached a local minimum on the potential energy surface, thus confirming the correctness of the model obtained through X-ray diffraction analysis.

### 6.2. Biological crystallography

The biomolecular sample states that we can study in the laboratory are (i) a crystal or fibre of pure molecules; (ii) a solution of non-aggregating pure molecules, perhaps in different 3D structural states; and (iii) single-particle pure complexes, again perhaps in different 3D structural states, on a cryoEM grid.

With those sample states we seek to understand the structural chemistry of a crowded, complex, mixture of biomol­ecules in the biological cell (Helliwell, 2020 ▸). This is a grand challenge. We have at the basis of biochemistry and molecular biology the quantum physics of atom-to-atom interactions and the movement of electrons and protons in chemical reactions. Can we ever hope to make trustworthy predictions in such complexities? Yet we do, and successfully so in cases such as pharmaceutical interventions. Those predictions are, of course, carefully assessed by multiple stages of clinical trials.

There are two ways of using the various methods available to biologists for studying these sample states. Firstly, we can integrate them to span the considerably different length scales of a single living system: nanometres, micrometres and millimetres upwards to metres. Secondly, within any given length scale we can combine methods, with their individual precisions, to get complementary views from each method and thereby achieve accuracy. A very powerful approach is a functional assay. In the biological example above, the colours of the lobster shell, the crustacyanin crystal and the solution (observed by eye and by UV–Vis spectroscopy) formed a powerful assay. Another type of assay could involve tracking an enzyme reaction from substrate to product with the appearance of the product monitored directly, *e.g.* by UV–Vis spectroscopy.

There are excellent textbooks describing the above, rather vast, topics. Peter Moore’s (2012 ▸) book is an excellent treatise spanning the whole topic of *Visualizing the Invisible: Imaging Techniques for the Structural Biologist*, including macromolecular crystallography, fibre diffraction and small-angle scattering, as well as optical microscopy and electron microscopy.

Chayen *et al.* (2010 ▸) provide a résumé of complementary techniques to macromolecular crystallography in their book from the perspective of protein crystal structure determination in structural genomics.

Even wider still is the vast compendium of the methods of molecular biophysics described in the book by Serdyuk *et al.* (2007 ▸), whose contents span mass spectrometry, thermodynamics, hydro­dynamics, optical spectroscopy, X-ray, neutron and electron diffraction, molecular dynamics, and NMR spectroscopy.

## 7. Examples in crystallography where trust broke down

Despite all of the above efforts, unfortunately, we have examples of malpractice in crystal structure analysis. We will not speculate on the motivations behind such behaviour. In the words of an editorial which appeared in *Nature Chemistry* (2011 ▸) ‘…we should acknowledge that scientific misconduct is happening, will always happen, and probably always has happened.’ Examples have occurred in both biological and chemical crystallography. In the case of biological crystallography, a high-profile case involved eleven individual protein crystal structures that were deemed to have probably been faked [Borrell (2009 ▸) provides a résumé]. Since then, the role of the PDB validation report, introduced in 2003, has been made essential to the peer review process in serious journals. The introduction of the *MolProbity* tool for biological macromolecules (Chen *et al.*, 2010 ▸) was another important step to establish the precision of reported structures.

In chemical crystallography, one of the most infamous examples of misconduct was the fabrication of a number of crystal structures published in *Acta Crystallographica Section E*, roughly between 2004 and 2011 (Harrison *et al.*, 2010 ▸; IUCr Editorial Office, 2011 ▸, 2012 ▸). The *modus operandi* employed for the fabrication involved the utilization of *bona fide* intensity data of correctly determined crystal structures reported in the literature to create new, fantasy structures. Three main strategies were used: (i) metal exchange in coordination complexes bearing the same ligand (*i.e.* the structure of a zinc complex would be use to obtain similar complexes with copper, cobalt, nickel *etc*.); (ii) element exchange in organic compounds (for instance, CH_2_ groups were replaced by NH_2_ or O and vice versa; OH groups were replaced with F atoms, and so on); (iii) both metal and element exchange in coordination compounds (especially with complexes of lanthanides). More recently, a preliminary report has drawn attention to the existence of a paper mill that has allegedly produced nearly 800 research papers on invented metal–organic frameworks endowed with supposedly therapeutic applications (https://doi.org/10.21203/rs.3.rs-1537438/v1). Many of these papers produced crystal structures that have been deposited in the Cambridge Structural Database. The staff at the Cambridge Crystallographic Data Centre are currently investigating the problem, and regular updates are available on their web site (https://www.ccdc.cam.ac.uk/support-and-resources/support/case/?caseid=819cfd76-c25d-40a2-ac9b-b4cf20d775a7).

These examples document the importance of data availability and reuse in pre- and post-publication peer review and assessment. At the same time, they also draw attention to the risks posed by over-manipulation of data (for example to fix problematic structures), which can also unintentionally lead to untrustworthy results. Whatever the case, even in the situation where a fabricated/modified crystal structure might still appear in a database and a publication, the availability of the underpinning data has led to improved checking procedures. *CheckCIF*, the validation tool routinely employed by IUCr Journals, and in general by crystallographers wanting to assess their structures, was introduced in 1998. Since then, it has been constantly updated, and a plethora of new tests and stringent criteria have been implemented (Spek, 2020 ▸). With these tools and the constant efforts of the scientific community, crystallography remains, notwithstanding, one of the scientific disciplines best equipped for detecting research misconduct (Clegg, 2021 ▸) and preventing or discovering scientific fabrication and/or incompetence.

## 8. Conclusions and future directions

Crystallography is a discipline where community-agreed processed diffraction data and model validation checks are routinely made. Although this system is not perfect, it provides the best chance for ensuring reliability and thereby trust in what we do.

The wider scientific scene has provided new insights on trust in science, such as FAIR and FACT. Within these more general considerations, it is also widely discussed that there is a general reproducibility crisis across the sciences and, although this has been rebutted in various ways, improvements in what scientists do are deemed to be possible (National Academies of Sciences, Engineering and Medicine, 2019 ▸). We suggest that conference education microsymposia could include these topics for presentation and discussion, with our Fig. 1 ▸ infographic as a guide to the topics to be included. Within this activity, crystallographers should debate the best way to answer possible public and student concerns about reproducibility and fabrication that may well arise in the future. The simplest answer we suggest is to demonstrate that depositing our raw, processed and derived data in a FAIR/FACT manner does greatly expose the ground truth of published conclusions and does allow scrutiny and test. We note and warmly welcome the journal *IUCrData*’s initiative launching a Raw Data Letters section of articles (https://iucrdata.iucr.org/x/services/journal_news.html).
